# Female University Students’ Physical Activity Levels and Associated Factors—A Cross-Sectional Study in Southwestern Saudi Arabia

**DOI:** 10.3390/ijerph10083502

**Published:** 2013-08-09

**Authors:** Atika Khalaf, Örjan Ekblom, Jan Kowalski, Vanja Berggren, Albert Westergren, Hazzaa Al-Hazzaa

**Affiliations:** 1 The PRO-CARE Group, School of Health and Society, Kristianstad University, SE-291 88 Kristianstad, Sweden; E-Mail: albert.westergren@hkr.se; 2 Department of Public Health Sciences, Karolinska Institute, SE-171 77 Stockholm, Sweden; E-Mail: vanja.berggren@ki.se; 3 Åstrand Laboratory of Work Physiology, The Swedish School of Sport and Health Sciences, SE-114 86 Stockholm, Sweden; E-Mail: orjan.ekblom@gih.se; 4 National Childhood Obesity Center, Departments of Pediatrics and of Clinical Science, Intervention, and Technology, Karolinska University Hospital, Karolinska Institute, SE-141 86 Stockholm, Sweden; E-Mail: jk@kowan.com; 5 Department of Health Sciences, Medical Faculty, Lund University, SE-221 00 Lund, Sweden; 6 Pediatric Exercise Physiology Research Laboratory, College of Education and Obesity Research Chair, King Saud University, Riyadh 11451, Saudi Arabia; E-Mail: alhazzaa@ksu.edu.sa

**Keywords:** epidemiologic methods, Middle East, women’s health, Saudi Arabia, physical activity, female university students, cross-sectional studies, socioeconomic factors

## Abstract

*Background*: The high prevalence of physical inactivity in Saudi Arabia is a growing challenge to public health. This study aimed to examine the prevalence of physical activity (PA) and associated factors among female university students. *Methods*: This cross-sectional study involved 663 randomly selected female university students who completed the Arab Teens Life Style questionnaire. Data included measurements of anthropometric, socioeconomic and environmental factors, as well as self-reported PA. Ordinal regression was used to identify associated factors with low, moderate and high PA levels. *Results*: The mean age of participants was 20.4 years (SD 1.5). Mean BMI of the students in relation to PA were 23.0, 22.9, 22.1 for high, moderate and low levels of activity, respectively. The analysis revealed significantly higher PA levels among married students, those with high educated mothers, and those who lived far from parks, and lower activity levels among underweight students. *Conclusions*: This study raises four important determinants for female university students’ PA levels. These factors could be of great importance in the endeavor to prevent the health-threatening increase in physical inactivity patterns and thus non-communicable diseases and obesity where the focus should be on the specific situation and needs of women in Saudi Arabia.

## 1. Introduction

The Kingdom of Saudi Arabia (KSA) has experienced a rapid progress in improving the health and well-being of its people during the past few decades [1]. One of the more significant consequences of the socioeconomic development in KSA has been lifestyle changes with subsequent adverse effects on health. In particular, an increase in obesity and physical inactivity has been evidenced [1,2,3]. The prevalence of physical inactivity in the Saudi society ranged from 43% to as high as 99% in certain segments of the population [3]. While women in KSA by traditional frequently engage in moderately intensive PA due to engagement in housekeeping tasks [3], they have among the lowest reported prevalence of moderate and vigorously intensive PA (2%) worldwide [4]. Furthermore, males reported more frequent participation in high intensity PA than their female counterparts [5], a finding that is consistent with global PA statistics [6].

Globally, the challenges of decreased PA levels are posing significant threats to the healthcare systems of many nations [7,8]. A significant increase in the level of physical inactivity has recently been reported on a global level [9]. Despite global concerns about the risk of individuals developing non-communicable diseases and/or becoming obese, PA surveillance and monitoring is carried out in relatively few countries [5].

In KSA, empirical studies have in recent decades started to focus on the healthy female population of KSA [10,11], reporting an alarming increase in physical inactivity and thus obesity and non-communicable diseases [12]. Similar studies have not been conducted on female university students in the southwestern part of KSA, despite the huge importance of studying the current situation of the females’ PA. Furthermore, the main determinants for physical inactivity among males and females in KSA was found to be marital status and education level, *i.e.*, spouses and people with lower levels of education were more likely to adopt an inactive lifestyle [2,3]. People with lower levels of PA tend to suffer from negative health consequences such as chronic cardiological and respiratory diseases, as well as unhealthy weight gain more often than their physically active peers [13]. The presence of several considerable health threats concomitant with low levels of PA suggest that physical inactivity should be one of the primary targets of obesity prevention in all age groups. 

The World Health Organization (WHO) has issued in 2010 recommendations for PA levels for promoting/maintaining health among adults [14]. They are based on the PA recommendations published by the U.S. Department of Health and Human Services [14]. The WHO PA recommendations are also similar to those of the American College of Sports Medicine (ACSM) [13]. WHO recommends aerobic PA of structured or unstructured character at moderate-intensity for 150 min, 75 min of vigorous-intensity aerobic PA or an equivalent combination of moderate-intensity and vigorous-intensity activity throughout the week as a means of health enhancement [13,14]. Moreover, according to WHO [15], there is a correlation between low PA, improper diet and overweight (OW)/obesity, yet, female public schools in KSA do not provide physical education to their students [16] despite general awareness of the positive effect regular PA has on the health of youth [8].

Despite the widely recognized benefits of PA [8,13,14] and the diversity of global recommendations published on PA [13,14], research on PA levels in female Saudi college students has been sparse, especially with respect to possible correlations with socio-cultural and environmental factors [16]. The aim of this study was to examine the prevalence of PA and associated socioeconomic and environmental factors among female university students in southwestern KSA.

The socioecological model is commonly used to recognize the interwoven relationship between the individual and his/her environment [17]. In this study, the socioecological model is used to explain the possible influence of socioeconomic factors on the PA levels of female university students in KSA.

## 2. Experimental Section

### 2.1. Design and Participants

A cross-sectional study design formed the basis of this study conducted among college-aged females at a university centre for women’s studies in southwestern KSA. The choice of this highly educated cohort of females allowed for a convenient sample that was eligible for completing the questionnaire in a study of a healthy Saudi Arabian female population with regard to their PA patterns. A statistical power of 80% with a 95% confidence interval (CI) was ascertained by selecting a sample of a minimum of 600 females among a total of 1,681 students. A sample selection with random multi-stage stratification was used. Thus, females were drawn on an equal basis from four levels (fresh-woman, sophomore, junior, and senior levels) at the university. Sampling insufficiency related to potential drop-out was precluded by distribution of 700 questionnaires which yielded 663 completed questionnaires during the spring of 2010. Three of the participants were subsequently eliminated during data analysis due to pregnancy. The study was approved by the Ethical Committee of King Khalid University, Abha, KSA (7/1078) and informed consent was obtained from all participants.

### 2.2. Anthropometric Measurements

Body mass (to the nearest 0.1 kg), body height (to the nearest cm) and waist circumference (to the nearest cm) were measured using a calibrated medical scale, stadiometer and measuring tape, respectively. BMI was calculated as body mass in kg over the squared height in meter. Waist to height ratio was calculated by dividing the waist measurement in cm by height measured in cm.

### 2.3. Instrument for Lifestyle Patterns

A questionnaire constituted the instrument of analysis in this study and it was a questionnaire built on the Arab Teens Life Style questionnaire (ATLS) [18,19]. Prior to the study, a pilot survey was conducted on a female university population of 30 participants to ensure that no modification was needed. The questionnaire included evaluations of anthropometric, socioeconomic, environmental, cultural factors, along with self-reported PA, sedentary activities and dietary habits. The PA questionnaire’s convergent validity was ascertained in relation to results of pedometric measurements made on youths aged 15–25 and showed a high reliability (intraclass correlation = 0.85; 95% CI: 0.70–0.93) and an acceptable validity (*r* = 0.30; *p* < 0.05) [18,19]. In a recent validity study on both males and females aged 14–19, the ATLS PA questionnaire was validated against the electronic pedometer and provided equivalent and significant coefficients for statistical surveying among Arabic youth (*r* = 0.37, *p* < 0.001) [20,21]. Diverse forms of PA were included in the questionnaire with regard to frequency, duration and intensity during a typical week. Energy expenditure in conjunction with household, leisure, transport, fitness and sports activities was taken into account. More specifically, PA measured in the survey included walking, stair climbing and descent, aerobic exercise, running, cycling, swimming, martial arts, calisthenics and resistance exercise, dancing, moderately intense sports like table tennis, badminton and volley ball, vigorously intense sports like basketball and handball, as well as household-related PA.

While intensity of activity or exercise during PA is measured in units of Metabolic Energy Turnover (MET), total activity volume is expressed as MET minutes per week resulting from a composite of PA time devoted to various MET activities [22]. The MET min/week is achieved by multiplying the intensity of the different activities (in METs) by time spent on the activity (in minutes/week).

Socioeconomic factors included information about age, marital status, educational level of the parents, number of siblings (sisters and brothers separately), type of residence and number of cars in the household. Environmental factors included the proximity between the participants’ residence and parks, malls and supermarkets. Furthermore, information was collected on the students’ PA-related behavior such as receiving parental support for regular exercise, reasons for regular and irregular exercise, respectively, and presence of regularly exercising parents and/or siblings. All socioeconomic and environmentally related background information was self-reported. In other words, the participants subjectively assessed the distances between their residence and parks, malls and supermarkets.

### 2.4. Statistical Analysis

Descriptive variables of prevalence and 95% CI were selected for this study where the response variable of PA levels was applied to METs tertiles of 220 participants in three groups characterized by low, moderate and high PA levels [23]. Tertile distribution enabled equivalency in intragroup comparisons and facilitated discernment of the association of PA to predictor variables. The following categories were identified for defining PA levels after the total sample was divided equally into three groups: low activity level (≤611.56 METs-min/week), moderately active (611.57 to 1,389.63 METs-min/week) and highly active (≥1,389.63). The descriptive data under Table 1 is presented in number of participants, row percentages, means and standard deviation (SD) when applicable. The row percentages were aimed to show the distribution of the predictor variables in percentages for the outcome variable of interest, namely Activity levels based on METs tertiles.

**Table 1:** Anthropometrics, and behavioral factors in relation to PA levels based on METs tertiles.

	Activity levels based on METs tertiles		
	Low, *n* = 220	Moderate, *n* = 220	High, *n* = 220
**Age**, mean (SD)	20.5 (1.5)	20.4 (1.4)	20.3 (1.5)
***Anthropometrics***			
**Waist to height ratio**, mean (SD)	45.1 (6.3)	45.7 (6.0)	45.5 (6.7)
**BMI**, mean (SD)	22.1 (4.3)	22.9 (4.0)	23.0 (4.4)
**BMI classification**, n (%)			
UW (BMI ≤ 18,49)	46 (47)	26 (26.5)	26 (26.5)
NW (BMI = 18.5–24.99) *	119 (31)	130 (35)	127 (34)
OW (BMI = 25–29.99)	32 (27)	45 (38)	42 (35)
Obesity (BMI ≥ 30)	11 (29)	11 (29)	16 (42)
***PA behaviors***			
**Meeting WHO recommendations of moderate-intensity PA (150 minutes/week)**, n (%)			
Yes *	18 (4)	177 (43)	217 (53)
No	202 (82)	43 (17)	3 (1)
**Meeting WHO recommendations of vigorous-intensity PA (75 minutes/week)**, n (%)			
Yes *	0 (0)	14 (14)	85 (86)
No	220 (39)	206 (37)	135 (24)
**Parents’ support for regular exercise**, n (%)			
Yes *	123 (32)	126 (32)	142 (36)
No	76 (35)	79 (37)	61 (28)
My parents discourage me from exercising	21 (40)	15 (28)	17 (32)
**Reasons for regular exercise**, n (%)			
For health *	80 (37)	73 (34)	61 (29)
To lose weight	58 (28)	77 (38)	69 (34)
To have fun	55 (31)	54 (31)	67 (38)
Other reasons	16 (36)	11 (25)	17 (39)
**Reasons for not exercising regularly**, n (%)			
Time scarcity *	123 (34)	115 (31)	130 (35)
Lack of a suitable place	44 (38)	41 (36)	30 (26)
No one is exercising with me	21 (31)	27 (40)	19 (29)
Other reasons	21 (41)	13 (26)	17 (33)
**Presence of regularly exercising parents or siblings**, n (%)			
Yes *	138 (32)	142 (33)	150 (35)
No	82 (36)	78 (34)	70 (30)

* Reference category for further statistical analysis. UW = underweight, NW = normal weight, OW = overweight. *n* = number of participants.

The presented variables for meeting the WHO recommendations of PA at moderate-intensity and vigorous-intensity levels, as presented in Table 1, were calculated by using two cut-off scores equivalent to moderate-intensity (150 min per week) PA and (75 min per week) of vigorous-intensity PA. The reasons for regular exercise with individually small numbers, namely To meet friends, For competition, and For other reasons, were all combined. Likewise, under the reasons for not exercising regularly, such as Lack of knowledge, Do not believe in the importance of exercise, Lack of healthy conditions and Other reasons were also combined.

Univariate and multivariate ordinal regression was employed to examine the association between activity levels and predictor variables. All significant (*p* < 0.05) and statistically indicated (*p* = 0.05–0.10) variables in the results of the univariate analysis were included in the multivariate regression analysis. At the final step, after finding a final multiple regression model, we tested all remaining non-significant variables one at a time to ensure that no other variables could significantly influence the final model. In the model for multivariate ordinal regression, the following variables were entered initially: classification of Body Mass Index (BMI), marital status, mother’s educational level and residential proximity to parks. For each predictor variable, a reference category was created. Alpha of 5% was selected for statistical significance determination, and statistical indication was defined within an alpha value of 5–10%. Odds ratio (OR) and confidence interval for OR were calculated in an Excel document, and the statistical analysis was run on IBM Statistics SPSS version 20 (SPSS Inc, White Plains, NY, USA). Analysis and interpretation of results were conducted in collaboration with a statistician (JK).

## 3. Results

Participants’ mean age was 20.4 years (SD 1.5) and their waist to height ratio was consistent (approximately 45%) for all groups. While the highly active students’ mean BMI was 23, those of the moderate and low activity level participants were 22.9 and 22.1, respectively. Underweight (UW) individuals most commonly displayed a low activity level (46%), and a substantial number of the OW/obese females were highly active (37%) (Table 1). 

With regard to the WHO recommendations for maintaining a moderate-intensity PA level, only 43% of the participants in the middle tertile met these guidelines, while 14% of the same group met the recommendations for vigorous-intensity activity (Table 1). Among the highly active females, only 1% failed to meet the WHO guidelines for moderate-intensity activity and 24% did not fulfill the requirements for vigorous-intensity PA. 

Of those whose parents did not encourage them to exercise regularly, 28% were highly active as compared with a 36% rate of high activity level among participants whose parents showed interest in their PA habits (Table 1). A majority of students whose aim was to lose weight through PA and who exercised regularly were moderately (38%) or highly (34%) active. Those exercising regularly as a means of health enhancement tended to display a low level of activity (37%) as compared with moderately active (34%) and highly active (29%) students. A considerable portion of the respondents exercised regularly for recreational purposes (low and moderate activity individuals 31%, and highly active 38%) or to lose weight (low active 28%, moderately active 38%, and highly active 34%). Time constraints, quoted as the main reason for students neglecting to exercise regularly, were indicated by 34% of those with a low level of activity, 31% of those who were moderately active and 35% of the highly active participants. Other main reasons were lack of a suitable place for exercise and lack of company to do the activities. Further, of those who had not regularly exercising parents or siblings 36% were low active, 34% were moderately active and 30% were highly active.

Socioeconomic characteristics, as detailed in Table 2 indicate the following: a majority of the participants were unmarried (33% of them were low active, 34% moderately active and 33% were highly active) and had two or more siblings; most of the respondents’ mothers were less educated than their fathers; a majority of the students belonged to households who owned three or more cars and lived in a villa. Most of the participants lived relatively close to supermarkets and had lived far from malls and parks (Table 2).

**Table 2:** Socioeconomic, and environmental factors in relation to PA levels based on METs tertiles.

	Activity levels based on METs tertiles		
	Low, *n* = 220	Moderate, *n* = 220	High, *n* = 220
***Socioeconomic factors***			
**Marital status**, n (%)			
Unmarried *	204 (33)	210 (34)	199 (33)
Married without children	7 (25)	6 (21)	15 (54)
Married with children	9 (47)	4 (21)	6 (32)
**Number of sisters**, n (%)			
None *	9 (37)	10 (42)	5 (21)
Only one	16 (29)	18 (33)	21 (38)
Two–three	67 (31)	79 (37)	69 (32)
Four–five	84 (38)	70 (32)	67 (30)
Six or more	43 (30)	43 (30)	58 (40)
**Number of brothers**, n (%)			
None *	5 (46)	3 (27)	3 (27)
Only one	21 (43)	12 (24)	16 (33)
Two–three	65 (28)	86 (37)	81 (35)
Four–five	75 (37)	61 (30)	65 (33)
Six or more	54 (33)	57 (34)	55 (33)
**Fathers’ level of education**, n (%)			
Primary or less *	42 (36)	36 (30)	40 (34)
Primary higher	43 (34)	47 (37)	36 (29)
Secondary	50 (33)	48 (32)	53 (35)
Bachelor	68 (33)	72 (35)	64 (32)
Master or PhD	17 (28)	17 (28)	27 (44)
**Mothers’ level of education**, n (%)			
Primary or less *	135 (38)	118 (33)	104 (29)
Primary higher	27 (23)	45 (38.5)	45 (38.5)
Secondary	32 (35)	30 (32)	31 (33)
Bachelor	23 (29)	23 (29)	34 (42)
Master or PhD	3 (23)	4 (31)	6 (46)
**Cars in the household**, n (%)			
No cars *	2 (67)	0 (0)	1 (33)
One car	22 (44)	14 (28)	14 (28)
Two cars	74 (37)	63 (31.5)	63 (31.5)
Three cars or more	122 (30)	143 (35)	142 (35)
**Type of residence**, n (%)			
Apartment *	33 (40)	24 (29)	25 (31)
Villa	126 (31)	125 (31)	153 (38)
One floor in villa	50 (33)	63 (42)	37 (25)
***Environmental factors***			
**Proximity to supermarkets**, n (%)			
Very close *	40 (28)	52 (37)	50 (35)
Kind of close	145 (37)	125 (31)	126 (32)
Far from house	35 (29)	43 (35)	44 (36)
**Proximity to malls**, n (%)			
Very close *	13 (46)	10 (36)	5 (18)
Kind of close	72 (29)	87 (35)	90 (36)
Far from house	135 (35)	123 (32)	125 (33)
**Proximity to parks**, n (%)			
Very close *	35 (36)	36 (37)	26 (27)
Kind of close	85 (39)	70 (32)	63 (29)
Far from house	100 (29)	114 (33)	130 (38)

* Reference category for further statistical analysis. *n* = number of participants.

**Figure 1:**
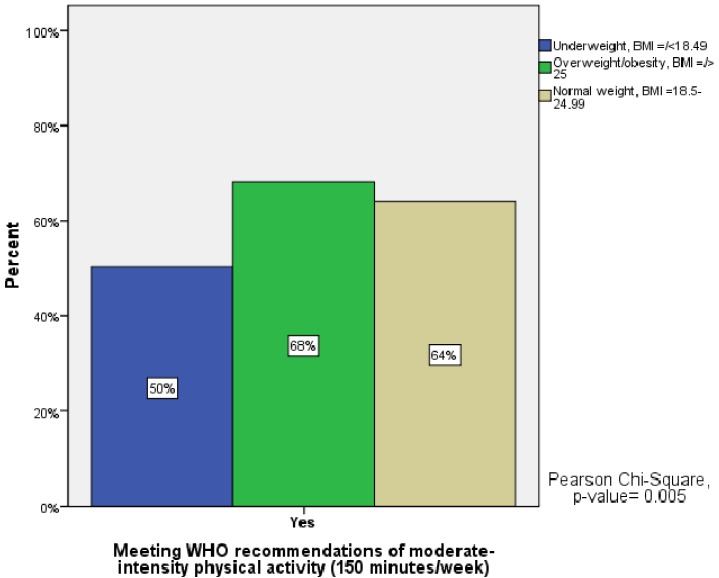
Meeting WHO recommendations of physical activity (PA) at a moderate-intensity level (150 min/week) in relation to underweight, normal weight and overweight/obese students.

Analysis of the respondents’ BMI categories with respect to the recommended amount of PA at a moderate-intensity level revealed that the proportion of students not meeting recommendations decreased significantly in each of the three BMI categories (UW = 50%, OW/obesity = 32%, and normal weight = 36%) (Figure 1).

**Figure 2:**
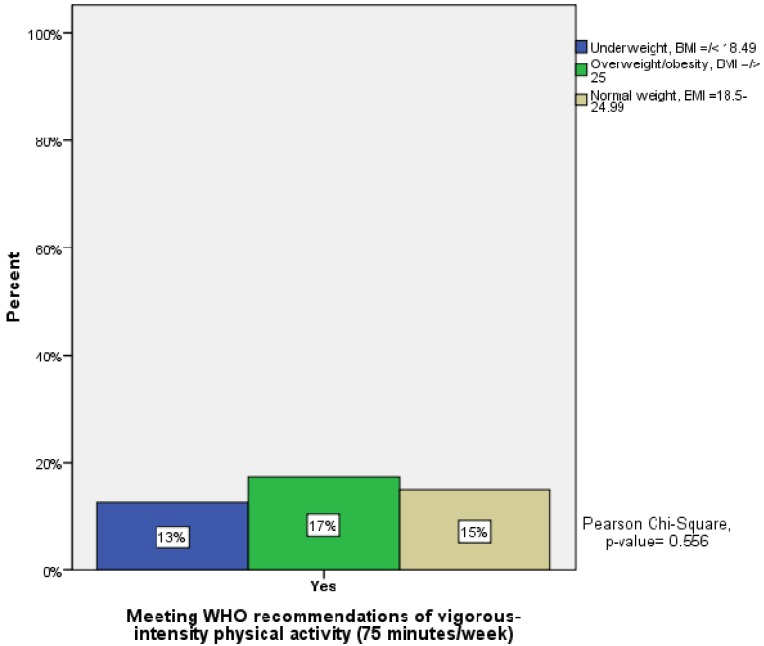
Meeting WHO recommendations of physical activity (PA) at a vigorous-intensity level (75 min/week) in relation to underweight, normal weight and overweight/obese students.

Furthermore, about 87% of the UW students did not meet the recommendations for vigorous-intensity activity levels compared to 85% of the normal weight (NW) respondents and 83% of those who were OW/obese (non-significant) (Figure 2).

Results from univariate analysis revealed a statistical indication (*p* = 0.05–0.10) that students who were not encouraged by parents to perform PA were less active (OR = 0.77, *p*-value = 0.092) than those who received encouragement (Table 3). When specified as the main reason for regular exercise, the desire to lose weight (OR = 1.38, *p*-value = 0.077) or to have fun (OR = 1.4, *p*-value = 0.06) were indicated as positive factors in PA habits. 

**Table 3:** Univariate ordinal regression analysis for the threshold of PA levels low, moderate and high (statistically indicated and significant variables are presented).

Variable	*n*	Estimate	Std. Error	*p*-value	OR	95% CI for OR
**Parents’ support for regular exercise**						
Yes *	391					
No	216	−0.26	0.16	0.092^ b)^	0.77	0.57–1.04
My parents don’t like exercising	53	−0.29	0.27	0.286	0.75	0.44–1.27
**Reasons for regular exercise**						
For health *	214					
To lose weight	204	0.32	0.18	0.077^ b)^	1.38	0.97–1.96
To have fun	176	0.36	0.19	0.056^ b)^	1.43	0.99–2.07
Other reasons	44	0.26	0.30	0.402	1.29	0.71–2.34
**Marital status**						
Not married *	613					
Married without children	28	0.73	0.37	0.047^a)^	2.07	1.01–4.24
Married with children	19	−0.37	0.43	0.386	0.69	0.30–1.60
**Mothers’ level of education**						
Primary or less *	357					
Primary higher	117	0.53	0.20	0.007^ a)^	1.70	1.16–2.51
Secondary	93	0.17	0.21	0.420	1.19	0.78–1.81
Bachelor	80	0.52	0.23	0.023^ a)^	1.68	1.07–2.63
Master or PhD	13	0.73	0.53	0.166	2.08	0.74–5.83
**Type of residence**						
Flat *	82					
Villa	404	0.38	0.22	0.088^ b)^	1.46	0.95–2.27
One floor in villa	150	0.04	0.25	0.863	1.04	0.64–1.72
Traditional house	23	−0.26	0.44	0.555	0.77	0.33–1.82
**Proximity to malls**						
Very close *	28					
Kind of close	249	0.80	0.38	0.034^ a)^	2.22	1.06–4.62
Far from house	383	0.58	0.37	0.118	1.78	0.86–3.65
**Proximity to parks**						
Very close *	97					
Kind of close	218	−0.03	0.23	0.907	0.97	0.63–1.51
Far from house	344	0.40	0.21	0.058	1.49	0.99–2.26
**BMI classification**						
NW (BMI = 18.5–24.99) *	376					
UW (BMI ≤ 18.49)	98	−0.54	0.21	0.011^ a)^	0.59	0.39–0.89
OW (BMI = 25–29.99)	119	0.14	0.19	0.480	1.15	0.79–1.68
Obesity (BMI ≥ 30)	38	0.26	0.31	0.406	1.30	0.70–2.40

* Reference category. UW = underweight, NW = normal weight, OW = overweight. ^a)^ Statistically significant, *p* < 0.05. ^b)^ Statistically indicated, *p* = 0.05–0.10. *n* = number of participants.

Statistical correlation was shown between married students without children and level of PA: these students were more than twice as much physically active (OR = 2.07, *p*-value = 0.047) than their unmarried counterparts. Moreover, a positive correlation was found between the mother’s level of education and PA (primary higher education, OR = 1.70, *p*-value = 0.007; bachelor degree, OR = 1.68, *p*-value = 0.023, compared to sub-primary level education), *i.e.*, the higher a mother’s level of education, the more active the student was.

Females residing in a villa were statistically indicated (OR = 1.46, *p*-value = 0.088) to be more active than those living in an apartment. The significance of proximity to malls as an indicator of PA level increased when the malls were located at an “acceptable” distance (OR = 2.22, *p*-value = 0.034), *i.e.*, fairly close and not too far away. Proximity to parks, however, was indicated positively in terms of its effect on PA if the parks were far away from the residence (OR = 1.49, *p*-value = 0.058) (Table 3). The participants’ BMI also showed a statistically significant relationship to PA levels, as UW persons had a negative correlation to PA (OR = 0.59, *p*-value = 0.011).

Table 4 shows results from a multivariate ordinal regression analysis for the thresholds of low, moderate and high PA levels. The analysis showed significantly higher PA levels among married students in comparison to unmarried (OR = 3.33, *p*-value = 0.005), those whose mothers had a higher primary education compared to less than primary education (OR = 1.89, *p*-value = 0.004), and those whose residence was further away from parks compared to parks close by (OR = 1.86, *p*-value = 0.009). The students with UW showed significantly less interest in PA compared to their NW counterparts (OR = 0.59, *p*-value = 0.018).

**Table 4:** Multivariate ordinal regression analysis for the threshold of PA levels low, moderate and high (only significant variables are presented).

Variable	Estimate	Std. Error	*p*-value	OR	95% CI for OR
**Marital status**					
Not married *					
Married without children	1.20	0.43	0.005^ a)^	3.33	1.45–7.64
Married with children	−0.73	0.47	0.121	0.48	0.19–1.21
**Mothers' level of education**					
Primary or less *					
Primary higher	0.64	0.22	0.004^ a)^	1.89	1.23–2.91
Secondary	0.27	0.25	0.274	1.31	0.81–2.12
Bachelor	0.29	0.26	0.263	1.33	0.81–2.19
Master or PhD	0.74	0.57	0.195	2.10	0.68–6.47
**Proximity to Parks**					
Very close *					
Kind of close	−0.00	0.25	0.997	1.00	0.61–1.64
Far from house	0.62	0.24	0.009^ a)^	1.86	1.17–2.96
**BMI classification**					
NW (BMI 18.5–24.99) *					
UW (BMI ≤ 18,49)	−0.53	0.22	0.018^ a)^	0.59	0.38–0.91
OW/obesity (BMI ≥ 25)	0.11	0.21	0.610	1.12	0.73–1.69

* Reference category. Pseudo R^2^ (Nagelkerke = 12.6%), model fitting (*p*-value = 0.000), Goodness-of-Fit (*p*-value = 0.132). ^a)^ Statistically significant, *p* < 0.05. UW = underweight, NW = normal weight, OW = overweight. Variables entered into the analysis: BMI classification, marital status, mothers’ level of education, proximity to parks and malls, reasons for regular and irregular exercise, parental support for exercising and type of residence.

## 4. Discussion

UW females were found to be less active than OW/obese students. Moreover, of those not encouraged by their parents to be physically active, only 28% were highly active. The mother’s level of education, the students’ marital status and residence proximity to parks all had a significant impact on the students’ PA.

The findings of this study with regard to PA levels among females are consistent with studies conducted in similar environments and cultures in other countries [24,25,26,27]. For example, the study by Al-Hazzaa, 2004 [23], showed a prevalence of physical inactivity levels that ranged between 43% and 99% among Saudi children and adults alike, in comparison to the present study that showed a high prevalence of students not meeting the WHO recommendations for PA at a vigorous-intensity level (85%). Results from other international studies conducted in different cultures with similar lifestyle patterns to that of the Kingdom of Saudi Arabia (KSA) [2,28] also indicated high inactivity levels among female students [29]. Reasons for the observed similarities may be explained in terms of a trend towards replacement of an active lifestyle with an increasing frequency of sedentary routines in daily life and a growing trend towards unhealthy weight gain. In addition, global physical inactivity patterns were reported to be more prevalent in affluent societies and among women [6,30]. This trend was also recently reported in KSA [31]. 

One of the more surprising findings in this study was the fact that UW females were found to be less active than their OW peers. We do not at present, however, have complementary data on the health status of the students who participated in the study and suggest further research in this area. Several explanations may be given for this apparent discrepancy: UW persons may display a lower level of PA relative to peers with greater BMI due to poor energy levels and/or a greater disposition to fatigue [32]. Another reason may be a wish from this group to increase their weight by being physically inactive [33]. Yet, this phenomenon has not been sufficiently studied earlier, and more research on the UW females’ PA patterns is crucial. Previous studies have reported a considerable decline in PA levels, especially among adolescent females in some of the major cities in KSA [23]. International studies, including that of Kimm *et al.* [30], have shown a significant age-specific decline in PA seemingly correlated with adolescence and the female sex. A recently published review study reported significant correlations between increased BMI and decline in PA levels [34]. Contrary to this, a study conducted on American university students of Arabic origin found that the OW students were more PA than their UW counterparts [35]. Levin *et al.*, [36] also reported that UW adolescents were less active than what previous studies had indicated. 

Another noteworthy finding of this study, however not statistically significant, was the proportion of highly physically active females (28%) who were not encouraged by their parents to exercise regularly, an observation that may at least in part be explained by the propensity of females to engage in household activities [37]. The extent to which parental influence impacts the PA patterns of young women has been examined in several studies and shown some or a considerable degree of correlation [38]. The students’ PA behaviors in relation to indicated effects of parental support of regular exercise can partially be explained by the socioecological model of Sallis and Owen [17]. In consistency with the socioecological model, the interpersonal factors seem to have an important role in shaping the students’ behavior with regard to PA levels. Although the students in the current study lacked a social network of physically active and/or supportive individuals, the students found ways to engage in high intensity PA. In addition, the results of this study confirm a positive and significant correlation between the mother’s level of education and the students’ PA levels. It may be argued that it is valuable to provide physical education classes for female students and some kind of intervention for their mothers. 

A number of evidence-based studies indicate that individuals who engage in PA at an adequate level are considered healthier than their physically inactive counterparts [4,5,6,7,8]. While recent indications that a large proportion of OW/obese females are physically active may be a sign that public health efforts and information in KSA have been effective, it may be too early to draw any conclusions of this nature. Messages from public health authorities with regard to the importance of PA are reaching the intended audience and seem to be affecting behavior patterns, but results have not yet been reported on any significant scale. Another possible reason for the reported high PA levels among OW/obese females could be the self-reported questionnaire that presents a risk for reporting the ideal levels of PA as reported in previous studies, according to Dumith *et al*. [27], instead of actual levels. Furthermore, a person’s perception of the ‘amount of effort required to perform an activity’ is also involved in her assessment of whether an activity should be characterized as being of low, moderate or high intensity. In the WHO definition of METs, intensity is the rate at which an activity is performed, or the effort required to perform the activity [14]. This may provide a partial explanation to why students with a high BMI showed relatively high levels of moderate-high PA. An activity that requires little effort for (some) students with low BMI may require a considerably greater effort on a subjective level for a “larger or heavier” student.

One of the possible limitations of this study may be related to the nature of the chosen instrument, *i.e.*, the self-reporting questionnaire based on the Arab Teens Life Style questionnaire (ATLS). Yet, this questionnaire was validated earlier with equivalent and significant validity coefficients for use among Arabic youth [21]. Furthermore, the questionnaire is comparable to other self-reporting instruments on the whole [39]. The results of this study should thus be transferable to other female university students, not only in KSA but also in other Arabic countries.

Another potential shortcoming of this study may be the rigor with which its results can be applied to expectations of PA behavior of the entire population in KSA, since the study participation was limited to female university students. In addition, these students live at a high altitude (2000–2300 m above sea level), which possibly makes the sample environmentally different compared to the rest of the KSA population. Nevertheless, the chosen sample was considered to be of representative size and with a statistical power of 80%, thus making the sample results transferable with respect to female university students, a strength in and of itself in the analysis.

Culturally fit research questions as a basis for motivation strategies regarding increasing PA levels may be one of the more important factors for achieving better health and adequate physical activity levels among the entire population of KSA, particularly among the more vulnerable groups such as females. The requirement to carry out a culturally adapted research method in this study was at least in part fulfilled by the choice and implementation of the adapted ATLS [21,22].

## 5. Conclusions

This study raises four important issues regarding female university students’ PA levels. These issues may be of great importance in the endeavor to prevent the health-threatening increase in physical inactivity and thus non-communicable diseases and obesity, where the focus should be on the specific situation and needs of women in KSA. As public health authorities in KSA continue their efforts to educate people on the importance of PA and a healthy lifestyle, the focus should be placed on providing support for community health centers. Efforts to improve the educational system, including the implementation of physical education classes for females should be made, and a larger number of public facilities for sports and exercise should be established specifically for women and adolescent girls. 

We recommend that ongoing studies be carried out to assess PA with more objective methods involving pedometer- or accelerometer-assessed levels of physical activity. Finally, we emphasize the need for intervention studies involving women’s PA.
